# Assembly and comparative analysis of the complete mitochondrial genome sequence of *Sophora japonica* ‘JinhuaiJ2’

**DOI:** 10.1371/journal.pone.0202485

**Published:** 2018-08-16

**Authors:** Yancai Shi, Yang Liu, Shouzhou Zhang, Rong Zou, Jianmin Tang, Weixue Mu, Yang Peng, Shanshan Dong

**Affiliations:** 1 Guangxi Institute of Botany, Chinese Academy of Sciences, Guilin, Guangxi, China; 2 Fairy Lake Botanical Garden, Shenzhen & Chinese Academy of Sciences, Shenzhen, Guangdong, China; 3 BGI-Shenzhen, Shenzhen, China; The National Orchid Conservation Center of China; The Orchid Conservation & Research Center of Shenzhen, CHINA

## Abstract

*Sophora japonica* L. (Faboideae, Leguminosae) is an important traditional Chinese herb with a long history of cultivation. Its flower buds and fruits contain abundant flavonoids, and therefore, the plants are cultivated for the industrial extraction of rutin. Here, we determined the complete nucleotide sequence of the mitochondrial genome of *S*. *japonica* ‘JinhuaiJ2’, the most widely planted variety in Guangxi region of China. The total length of the mtDNA sequence is 484,916 bp, with a GC content of 45.4%. *Sophora japonica* mtDNA harbors 32 known protein-coding genes, 17 tRNA genes, and three rRNA genes with 17 *cis*-spliced and five *trans*-spliced introns disrupting eight protein-coding genes. The gene coding and intron regions, and intergenic spacers account for 7.5%, 5.8% and 86.7% of the genome, respectively. The gene profile of *S*. *japonica* mitogenome differs from that of the other Faboideae species by only one or two gene gains or losses. Four of the 17 cis-spliced introns showed distinct length variations in the Faboideae, which could be attributed to the homologous recombination of the short repeats measuring a few bases located precisely at the edges of the putative deletions. This reflects the importance of small repeats in the sequence evolution in Faboideae mitogenomes. Repeated sequences of *S*. *japonica* mitogenome are mainly composed of small repeats, with only 20 medium-sized repeats, and one large repeat, adding up to 4% of its mitogenome length. Among the 25 pseudogene fragments detected in the intergenic spacer regions, the two largest ones and their corresponding functional gene copies located in two different sets of medium-sized repeats, point to their origins from homologous recombinations. As we further observed the recombined reads associated with the longest repeats of 2,160 bp with the PacBio long read data set of just 15 × in depth, repeat mediated homologous recombinations may play important role in the mitogenomic evolution of *S*. *japonica*. Our study provides insightful knowledge to the genetic background of this important herb species and the mitogenomic evolution in the Faboideae species.

## Introduction

Plant mitochondrial (mt) genomes differ from their animal counterparts mainly by the considerable expansion of the non-coding regions and dynamic genome structure induced by repeat-mediated homologous recombination [1]. Angiosperm mt genomes show a distinctive 200-fold size divergence ranging from 66 Kb in *Viscum scurruloideum* [2] to 11.3 Mb in *Silene conica* [3], which could be primarily attributed to the expansion or contraction of the intergenic sequences of intracellular gene transfers [4], horizontal gene transfers [5], and species-specific sequences of unknown origin [6]. As an important component of the non-coding regions of angiosperm mitogenomes, repetitive sequences play essential role in shaping and maintaining plant mitogenomic structure [7, 8] via participation in genome rearrangements [9], recombination dependent replication initiations [10], genome sequence duplications, inversions, insertions, and deletions [11]. Homologous recombination appears to be positively correlated with repeat length [2]: large repeats (≥1,000 bp) are frequently reported to cause recombination equilibrium [12], whereas medium-sized (100–1000 bp) and small (<100 bp) repeats tend to mediate moderate- to- minor recombinations [7, 13]. Repeat identity is another important factor related to recombination activity as has been suggested by findings in several *Silene* species [13]. On one hand, higher sequence similarity can facilitate recombination, but on the other hand, frequent recombination can homogenize repeat copies more frequently throughout the genome [13].

High throughput sequencing technology has greatly enhanced the research of plant mt genome. There are (as of May 2018) 214 complete mt genomes sequenced and deposited in GenBank organelle genome database (https://www.ncbi.nlm.nih.gov/genome/organelle/). Ninety-six of these belong to 27 angiosperm orders with a strong bias towards crops, such as 15 members of the Poales, 11 Brassicales, 11 Malvales, and 8 Solanales, and provides good opportunities for comparative mitogenomic studies in these economically important crop groups. Studies have revealed evolutionary rearrangement mechanism in *Brassia* [14], highly heterogeneous substitution rates and its implication for genome size evolution in *Silene* [13]. Fabaceae (Fabales) is the third largest family of flowering plants with 751 genera and 19,500 described species [15]. This family includes a number of important agricultural food plants [16], such as *Glycine max* and *Vigna radiata*. There are currently seven mt genomes available for Fabaceae that belong to four tribes (Phaseoleae, Millettieae, Loteae, and Trifolieae) of the subfamily Faboideae, including *Medicago truncatula* (GenBank Accession number: KT971339), *Lotus japonicus* [17], *Millettia pinnata* [17], *G*. *max* [18], *V*. *angularis* [19], *V*. *radiata* [20], and *V*. *radiata* var. *radiata* [21]. These Faboideae mt genomes range from 271 Kb in *M*. *truncatula* to 425 Kb in *M*. *pinnata* while holding a similarly reduced gene set with three ribosomal protein genes (*rps2*, *rps11* and *rps13*) completely lost and two protein genes (*rps19* and *sdh4*) pseudogenized. Some Faboideae mt genomes retained pseudogene fragments of *rpl2*, and/or *rpl5*, and/or *rps7*, and/or *sdh3*, suggesting either slow pseudogene deletion rates or recent transfers to nuclear genome as in the study of the recent intracellular gene transfer of *cox2* gene between mitochondrion and nucleus in Legumes [9]. Besides, some Faboideae mt genomes are highly repetitive, such as *G*. *max* with 150 repeats producing a molecular pool of 760 circles via active repeat recombinations [18]. However, other mt genomes are depauperate in repeats, such as *V*. *radiata*, with only 62 small- and medium-sized repeats mediating minor or no recombination [20]. Comparative analysis of the mitogenomes in the Faboideae would provide new insight into the mitogenomic evolution and phylogenetics of the Faboideae.

*Sophora japonica* L. (Faboideae, Leguminosae), known as Chinese Scholar Tree, is a well-known traditional Chinese herb with a long history of cultivation [22]. Its flower buds and fruits, with high content of a variety of flavonoids [23], are used as a hemostatic agent in traditional Chinese medicine [22] and therefore, widely used in industrial extraction [24] of an active pharmaceutical ingredient, rutin, which is frequently reported to exert positive effects in animal body metabolism, including anti-platelet [25], antioxidant [26], and anti-inflammatory [27]. The highest rutin content (25%–40%) was observed in *S*. *japonica* ‘Jinhuai’, which is mainly planted in the Northern region of Guangxi province, China [28]. Molecular studies of this important herb species will provide insightful information into its genetic background. Here, we report the complete mt genome of the most widely planted cultivar of *S*. *japonica* ‘JinhuaiJ2’ based on a combined approach employing Illumina Next Generation Sequencing and PacBio SMRT sequencing technologies.

Our study recovered a high quality mt genome assembly of *S*. *japonica* with a length of 484,916 bp, which at present is the largest in the Faboideae, primarily because of the expansion of species-specific intergenic spacer regions. The genome encodes 52 genes with 32 slow evolving protein coding genes, three rRNA genes, and 17 tRNA genes. These genes are largely conserved in the Faboideae with the exception of three protein coding genes (*cox2*, *rps1*, and *sdh3*), and two tRNAs (*trnSgct* and *trnTtgt*). *S*. *japonica* mitogenome has a low proportion of repeated sequences and resembles that of the *V*. *radiata* and *M*. *truncatula*. However, we observed pseudogene fragments in the intergenic spacers associated with the repeats, along with the recombined reads for the longest repeats of 2,160 bp. The significance of repeat activities in the mitogenomic evolution of species in the Faboideae has also been exemplified in small repeats induced intron length variations, and dynamic mitogenome rearrangements.

## Materials and methods

### DNA extraction and sequencing

Total genomic DNA was extracted using the CTAB method [29] from five grams of fresh leaves harvested from a single tree of *S*. *japonica* ‘JinhuaiJ2’ at Gui Lin Botanical Garden, Chinese Academy of Sciences, in Guilin, and a voucher specimen has been deposited in the Herbarium of Guangxi Institute of Botany (IBK: collection number *Shi 20170630–1*). PacBio SMRT sequencing and Illumina Next Generation Sequencing (NGS) were carried-out at the Benagen Ltd. (Wuhan, China). For Illumina HiSeq 2000 platform, 1μg high quality genomic DNA was sheared into 300–500 bp fragments using Covaris M220, and subsequently used to generate the sequenced library using TruSeq^TM^ DNA Sample Prep Kit following the manufacturer’s instructions. The library was amplified using TruSeq PE Cluster Kit v3-cBot-HS and sequenced using TruSeq SBS Kit v3-HS for 200 cycles, and yielded 5,066 Mb sequencing data of 150 bp paired-end (PE) reads. For the PacBio RSII platform, the enriched organellar DNA of *S*. *japonica* ‘JinhuaiJ2’ was isolated according Richardson et al. [30]. A total of 10 μg high quality DNA was sheared into 8–10K fragments with a Coveris gTube and sequencing was performed for a single cell. The raw PacBio reads were blasted against the plant mitogenomic data base downloaded from NCBI Organelle Genome Resources website (https://www.ncbi.nlm.nih.gov/genome/organelle/) for the mining of the PacBio mt reads for subsequent analysis.

### Genome assembly and annotation

**Fig 1:**
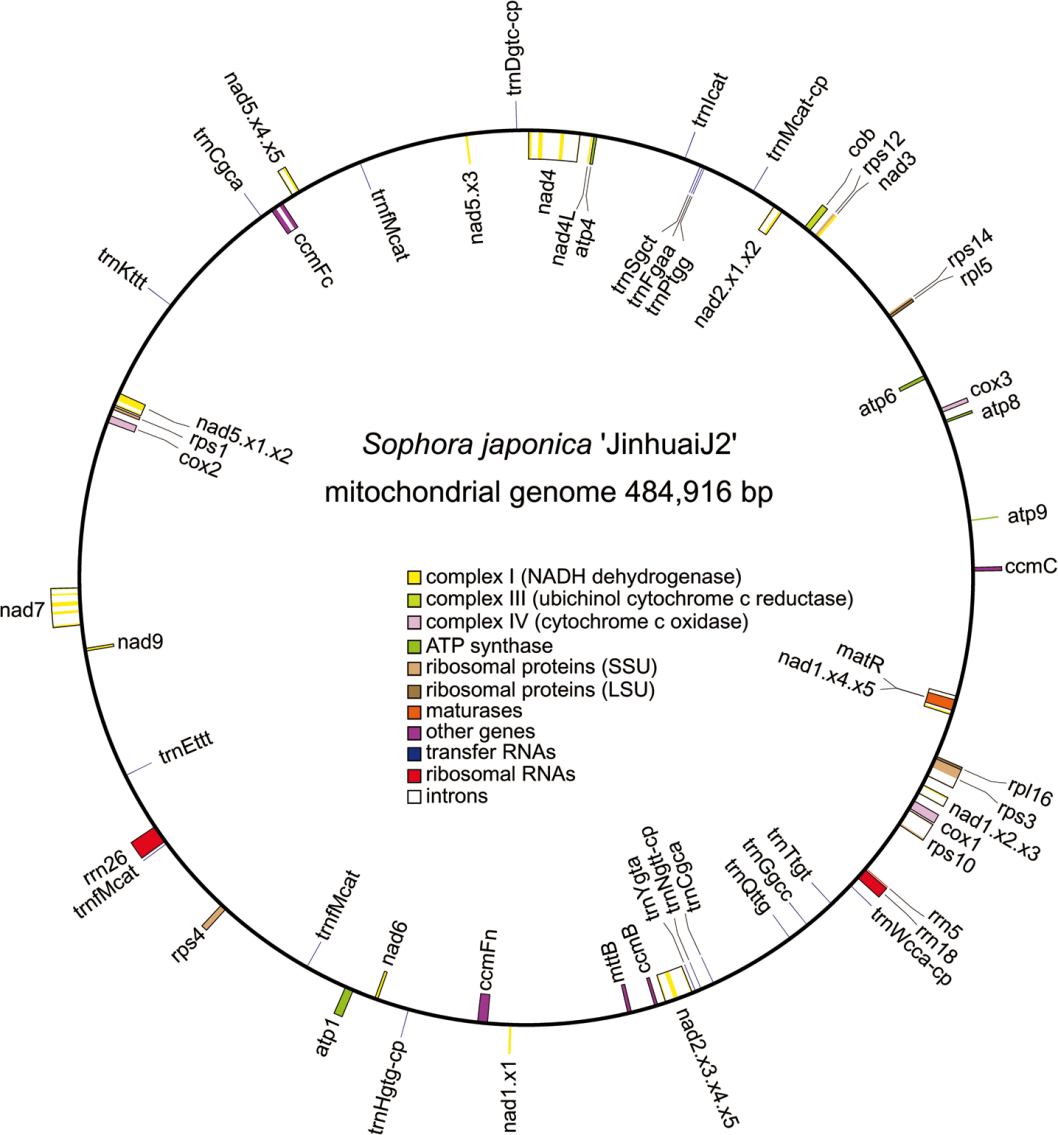
Mitochondrial genome map of the master circle configuration of *Sophora japonica* ‘JinhuaiJ2’. Genes (exons are closed boxes) shown on the outside of the circle are transcribed clockwise, whereas those on the inside are transcribed counter-clockwise. Genes from the same protein complex are colored the same, introns are indicated in white boxes, and tRNAs of chloroplast origin are noted with a ‘-cp’ suffix.

The raw Illumina PE read data in fastq format were trimmed and filtered for adaptors, and low quality reads, undersized inserts, and duplicate reads eliminated using Trimmomatic [31]. A clean Illumina PE read data set of 4,591 Mb was produced for subsequent PacBio mt read correction and mitogenome assembly validation (S1 Table). The 1,104 mitochondrial PacBio long reads (total bases: 15,884,959 bp; average length: 14,389 bp) were corrected using LoRDEC [32] with the clean Illumina PE read data to an accuracy of 99%. Genome assembly was carried-out using the program Canu v1.7 [33], which yielded a mitochondrial contig of 484,916 bp with an average read depth of 15×. The clean PE reads were then mapped to the recovered mt contig to further validate the genome assembly, which yielded a circular genome of 484,916 bp (Fig 1; GenBank accession number: MG757109).

The *S*. *japonica* mitogenome was annotated following the steps as described in Xue et al [34]. In summary, protein-coding and rRNA genes were annotated by blastn searches of the non-redundant database of National Center for Biotechnology Information (NCBI). The exact gene and exon/intron boundaries were further confirmed in Geneious v10.0.2 (www.geneious.com) by aligning orthologous genes from available annotated land plant mt genomes to those of *S*. *japonica*. The tRNA genes were annotated using tRNAscan-SE v2.0 [35]. The mtDNA sharing was estimated following Guo et al. [36] using Mauve 2.3.1 as implemented in Geneious 10.0.2. The known sequence content of each Faboideae mitogenome was ascertained by searching the mitogenome sequence against the NCBI nucleotide data base, and the corresponding anonymous sequence content was calculated by subtraction of the known sequence content from the total genome length. Putative RNA editing sites were predicted using Blastx prediction as implemented in the software PREPACT2 (http://www.prepact.de/prepact-main.php) with default blast options. The protein gene references were sampled from mitogenomes of *L*. *japonicus* and *M*. *pinnata*, respectively. The genome map of *S*. *japonica* was generated by the online program OGDRAWv1.2 [37].

### Phylogenetic reconstruction and substitution rate estimation

**Fig 2:**
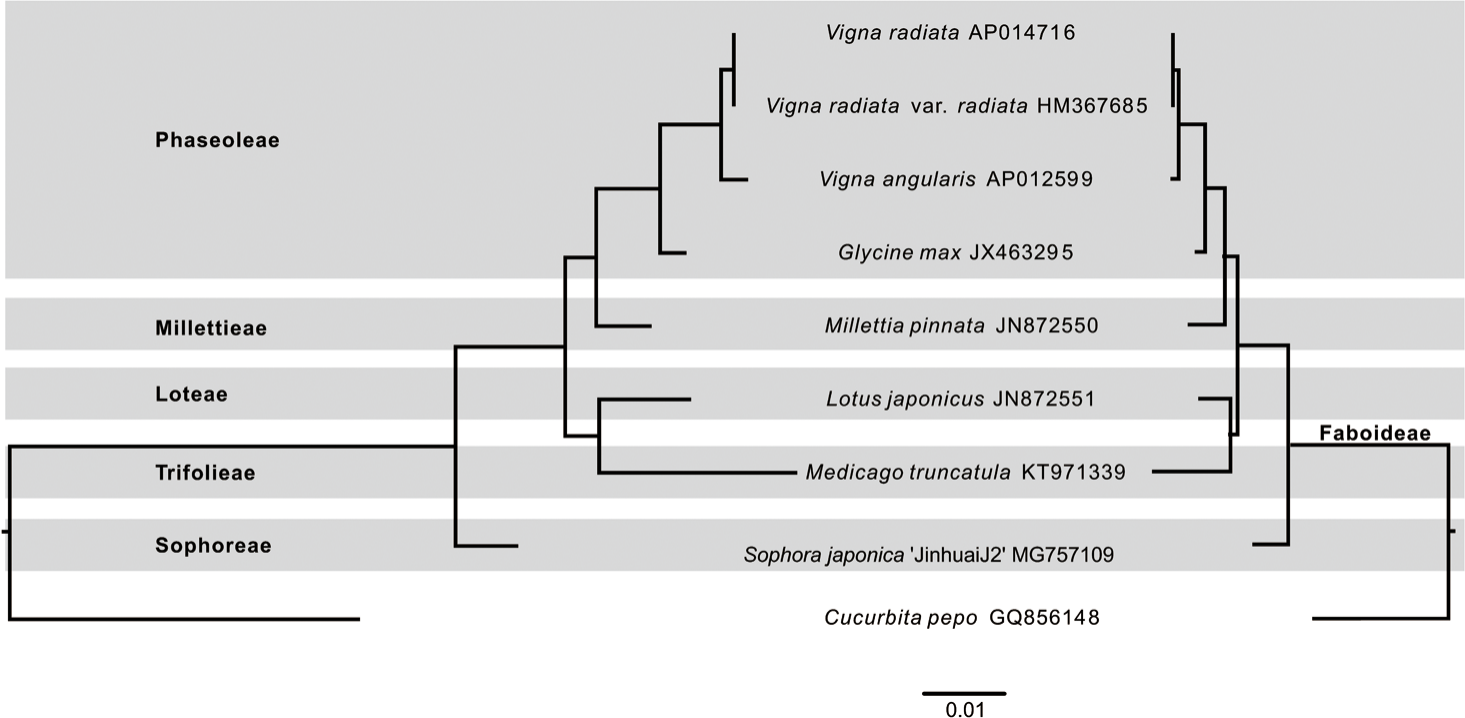
Nucleotide substitution rates in eight Faboideae species with fully sequenced mitochondrial genomes. Branch lengths are proportional to rates of synonymous (left panel) and nonsynonymous (right panel) substitutions based on a concatenated alignments of 29 protein genes, and with the tree topology based on amino acid sequences from 29 protein coding genes.

Phylogenetic analyses were performed using both nucleotide and amino acid sequences from 29 protein-coding genes (*atp1*, *atp4*, *atp6*, *atp8*, *atp9*, *ccmB*, *ccmC*, *ccmFc*, *ccmFn*, *cob*, *cox1*, *cox2*, *cox3*, *nad1*, *nad2*, *nad3*, *nad4*, *nad4L*, *nad5*, *nad6*, *nad7*, *nad9*, *rpl5*, *rpl16*, *rps3*, *rps4*, *rps10*, *rps12*, *rps14*) of the nine complete mt genomes (*Cucurbita pepo* GQ856148, *S*. *japonica* ‘JinhuaiJ2’ MG757109, *M*. *truncatula* KT971339, *L*. *japonicus* JN872551, *M*. *pinnata* JN872550, *G*. *max* JX463295, *V*. *angularis* AP012599, *V*. *radiata* HM367685, and *V*. *radiata* var. *radiata* AP014716). The nucleotide and amino acid sequences were extracted from the nine mitogenomes using a local Perl script, and aligned using the software Mafft v5 [38], respectively. The resulting alignments were optimized using the online program Gblocks (http://molevol.cmima.csic.es/castresana/Gblocks_server.html). Filtered alignments were concatenated using a local Perl script, which generated a final alignment of 8,635 bp for the amino acid data set, and 25,950 bp for the nucleotide data set, respectively. The phylogenetic reconstruction was carried-out in raxmlGUI [39] using Poisson model with the final amino acid sequence alignment. The bootstrap consensus tree was inferred from 1,000 replicates using *C*. *pepo* as the outgroup. All the nodes were supported by 100% bootstrap values with the exception of the node consisting of *M*. *truncatula* and *L*. *japonicus* (S1 Fig). The phylogenetic tree (Fig 2) inferred from a concatenated data set of 29 amino acid sequences was congruent with the placement of Legume Phylogeny Working Group [40]. The substitution rates were calculated using the program Hyphy v2.2 [41] with a local codon model, the MG94×HKY85_3×4 model with the nucleotide sequence alignment and the best tree generated from the above-mentioned amino acid alignment. The branch age was estimated according to TimeTree website (http://www.timetree.org/). Absolute rates of substitution were calculated by dividing the Hyphy branch substitution rates by the branch age for each branch in the tree following Richardson et al [30].

### Repeat identification and genome structure analysis

Repeated sequences of all the eight Faboideae mt genomes were identified according to Guo et al. [36] by blasting each of the mitogenomes against itself with an e-value cut-off of 1e^-6^, and a word size of seven. The blastn output was parsed using a local Perl script to identify the repeat pairs with unique start and end coordinates. The repeats were classified into the following three groups: large (≥1000 bp), medium (100–1000 bp) and small repeats (<100 bp). The repeat distribution map on the genome and comparisons for abundances and occurrences were generated using Circos v0.67–7 [42]. The gene orders of the eight Faboideae mt genomes were extracted using a local Perl script and compared with each other using UniMoG [43] to identity gene rearrangements among these genomes. The pair wise genome rearrangement times were imported into a tab delimited data matrix to generate a heatmap using ggplot2 [44] as implemented in R v3.3.0 (https://www.r-project.org/). The cladogram, based on the amino acid sequences of 29 protein-coding genes of the eight Faboideae mt genomes, was constrained according to version 13 of the Angiosperm phylogeny website (http://www.mobot.org/mobot/research/apweb/) following Guo et al [36]. The divergence time on the tree was estimated from Timetree website (http://www.timetree.org) following Richardson et al [30].

## Results and discussion

### Genome features of the mitogenome of *S*. *japonica*

**Fig 3:**
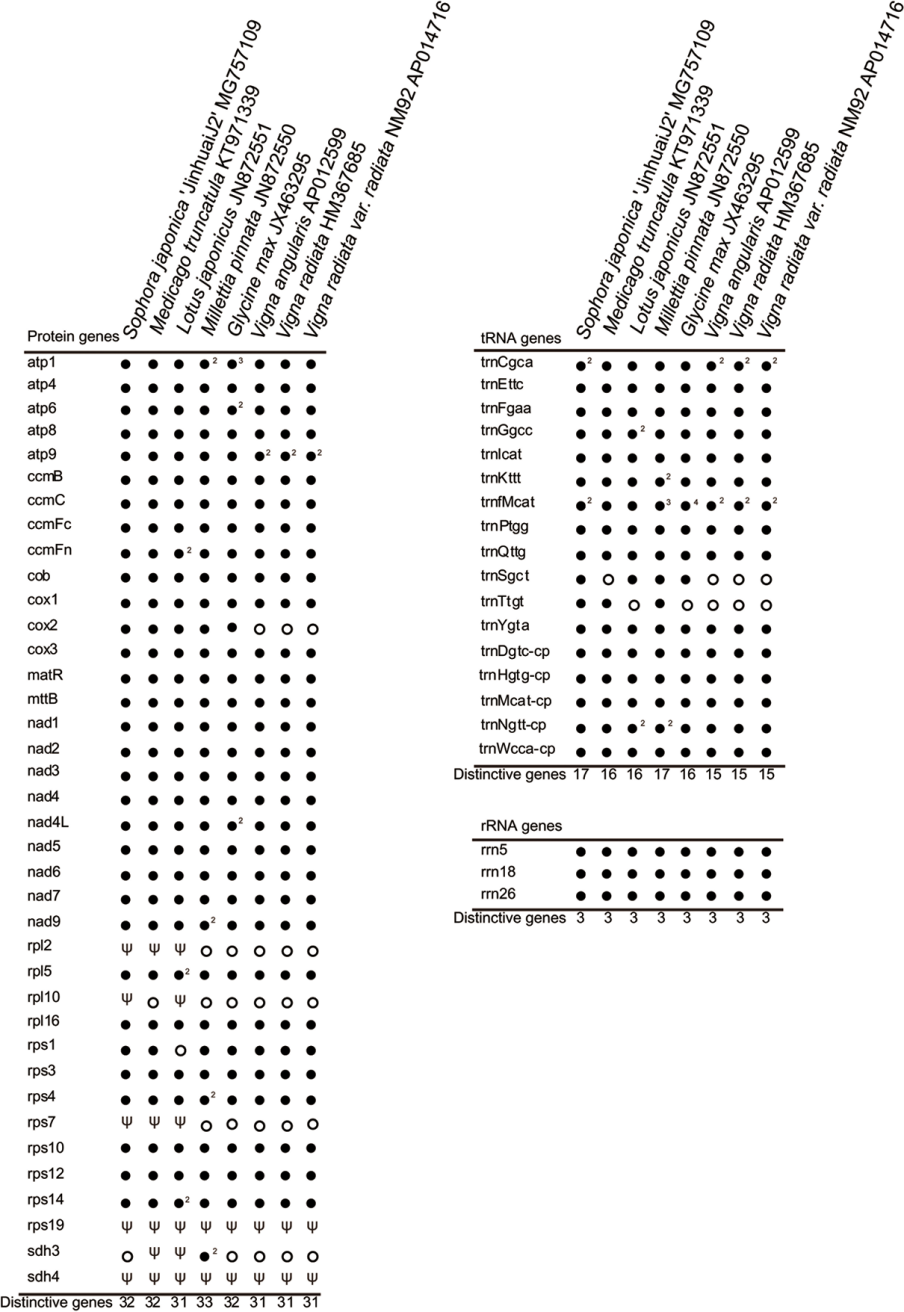
Gene content of eight Faboideae mitogenomes. The solid dots (●) indicate the presence of a gene, and the open dots (°) their absence. The symbol “ψ” indicates the presence of a putative pseudogene. The digit-superscripts indicate the copy number of the specific genes. For each species, and the total numbers of distinctive intact genes summarized at the bottom.

The mt genome of *S*. *japonica* was assembled into a single circular molecule of 484,916 bp (Fig 1), and is larger than all the other seven mt genomes reported from the Faboideae, which range from 271,618 bp in *M*. *truncatula* to 425,718 bp in *M*. *pinnata*. The relatively large size of the mt genome of *S*. *japonica* is primarily due to the accumulation of species-specific noncoding sequences, especially the intergenic spacer expansions, which consist of 420,569 bp, or 86.7% of the mitogenome of *S*. *japonica*. The mitogenome encodes 32 known protein-coding genes, three rRNA genes, 12 native mitochondrial tRNA genes, and five chloroplast derived tRNA genes (Table 1, Fig 3). None of the protein genes in the mitogenome of *S*. *japonica* retained duplicated copies, resembling the observation *M*. *truncatula* with a significantly reduced genome size. Protein coding sequences comprised only 6.1% (29,501 bp) of the total length of the mitogenome of *S*. *japonica*, which is 4.5 Kb shorter than *G*. *max* and *M*. *pinnata* that has three or four protein genes either duplicated or triplied. The *S*. *japonica* mitogenome possesses a 17 tRNA gene set identical to that of the *M*. *pinnata*, whereas it differs from the rest of the Faboideae mt genomes by the presence of two tRNAs (*trnSgct* and *TrnTtgt*) that are either present in others or absent. In general, the gene content of *S*. *japonica* is very similar to the other published mt genomes of the Faboideae, especially *M*. *pinnata*. The gene set of *S*. *japonica* differs from that of the *G*. *max* and *M*. *truncatula* by containing *trnTtgt* and *trnSgct*, and from *V*. *radiata* and *L*. *japonicus* by its presence of *cox2* and *rps1*, respectively.

**Table 1:** Comparison of mitochondrial genome traits in Faboideae^a^.

Genome feature^a^	Sja	Mtr	Lja	Mpi	Gma	Van	VraI/ VraII
Accession	MG757109	KT971339	JN872551	JN872550	JX463295	AP012599	HM367685/ AP014716
Size (bp)	484,916	271,618	380,861	425,718	402,558	404,466	401,262
GC(%)	45.4	45.4	45.4	45.0	45.0	45.2	45.1
Genes	53	52	51	54	52	50	50
tRNAs	18	17	17	18	17	16	16
rRNAs	3	3	3	3	3	3	3
Protein genes	32	32	31	33	32	31	31
RNA Editing sites	ca. 480	ca. 436	528	510	ca. 514	ca. 461	ca. 468
Repeats^b^ (bp)	19,601	5,406	57,409	58,847	72,873	13,451	7,925
Genes (bp)	64,347	60,179	67,655	76,077	73,265	65,756	66,095
Protein exons (bp)	29,501	28,435	31,195	34,012	34,133	28,918	28,879
Intron (bp)	28,035	25,209	29,940	35,119	32,553	30,398	30,772

Notes

^a^ (Sja) *Sophora japonica* ‘JinhuaiJ2’; (Mtr) *Medicago truncatula*; (Lja) *Lotus japonica*; (Mpi) *Millettia pinnata*; (Gma) *Glycine max*; (Van) *Vigna angularis*; (VraI) *V*. *radiata*; (VraII) *V*. *radiata var*. *radiata*.

^b^ Repeats with length > 19 bp and identity >80.

**Fig 4:**
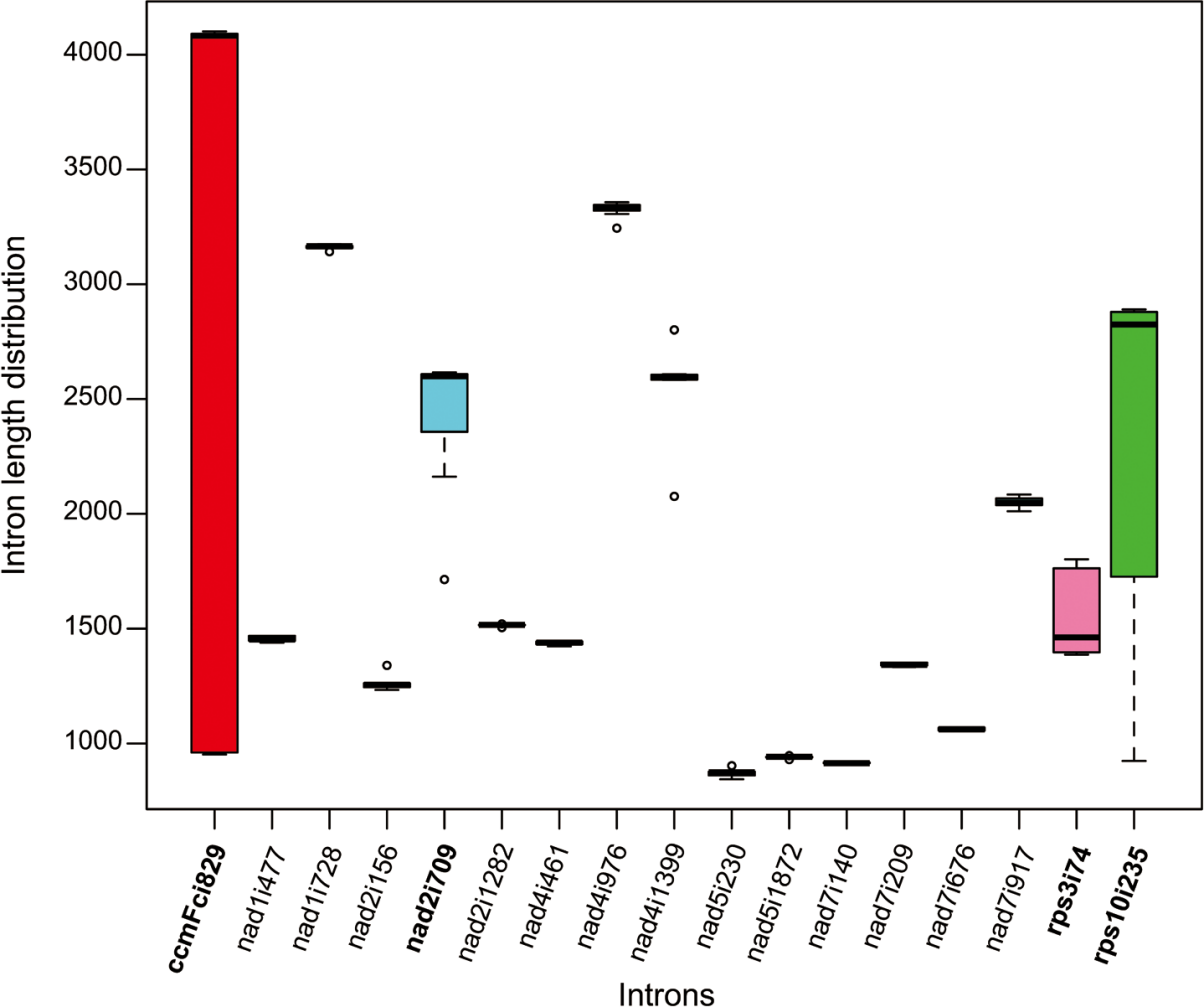
Boxplot diagram showing 17 cis-spliced intron length distributions across eight Faboideae mitogenomes.

**Fig 5:**
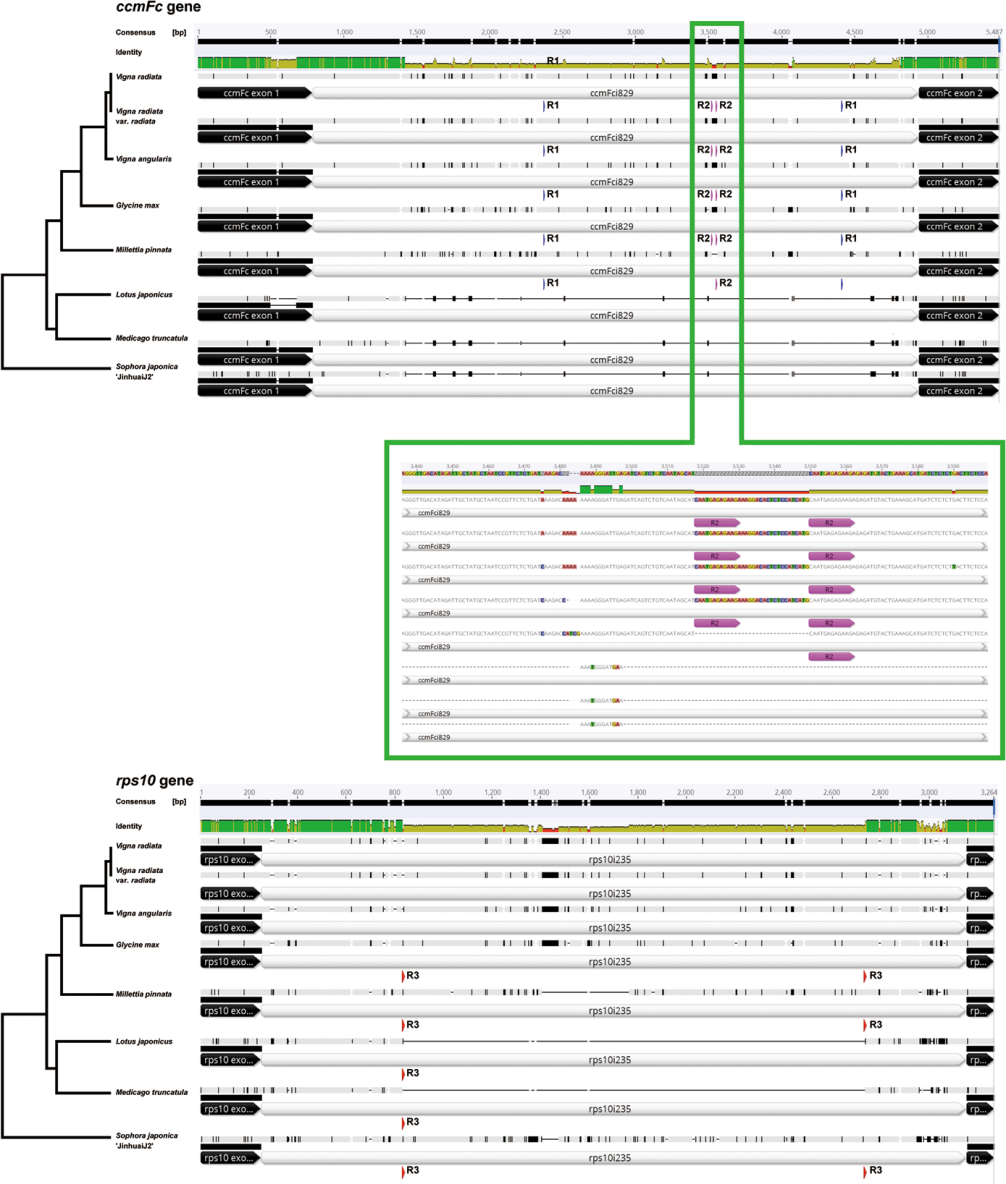
Schematic demonstration of the two protein gene alignments (*ccmFc* and *rps10*) showing intron length variations and the positions of the intron-positioned direct repeats.

The *S*. *japonica* mitogenome contains 22 group II introns, including 17 cis-spliced and five trans-spliced introns disrupting eight protein-coding genes (*ccmFc*, *nad1*, *nad2*, *nad4*, *nad5*, *nad7*, *rps3*, and *rps10*). Similar intron sets were also present in seven Faboideae mt genomes. However, the total intron length in the Faboideae varied significantly, and ranged from 25 Kb in *M*. *truncatula* to 35 Kb in *M*. *pinnata* (S2 Table). All of the intron alignments were examined and four (*ccmFci829*, *rps10i235*, *rps3i74*, and *nad2i709*) out of 17 cis-spliced introns showed distinct intron length variations (Fig 4). Some of these intron deletions may be induced by the activity of small repeats that only measured a few bases (Fig 5; S2 Fig). Intron *ccmFci829* of the mt genomes of *S*. *japonica*, *M*. *truncatula* and *L*. *japonicus* measured only 950 bp, which is about 3 Kb shorter than that of the rest of the Faboideae with 4 Kb. NCBI blastn searches of these intron sequences against nucleotide data base identified the 950-bp *ccmFci829* as the ancestral type because of its continuous distribution in eudicot mitogenomes. The 4-Kb *ccmFci829* intron is a derived type with distributions limited in some Faboideae mt genomes, and the unique 3-Kb region inserted returned no blast hits, which indicated anonymous sequence insertions in the common ancestor of Millettieae and Phaseoleae at some evolutionary stage. Two sets of short direct repeats were detected in the intron region of *ccmFci829*, and included the 15 bp repeat R1, and the 13 bp repeat R2. The activity of R1 could lead to the deletion of its flanking region of 2 Kb. Although the deleted form was not detected in the four mt genomes examined here, it is possible that extended sampling of this intron region in the Faboideae could provide more insights into variation mechanisms of intron length. The activity of repeat R2 could lead to the deletion of its 19 bp flanking region, and this deleted type was observed in *M*. *pinnata*. Intron *rps10i235* also showed distinct intron length variations exclusively in the Faboideae with possibly recent mitovirus sequence insertion [18] in all the five species except *L*. *japonicus* and *M*. *truncatula*. These two species maintained uniform *rps10i235* introns with a length commonly seen in eudicots, which indicated either the secondary loss of the insertions in the two species or parallel gains of the insertion in the other three tribes of the Faboideae. Considering the position of the repeat R3, which is precisely located at the edge of the deletions of *rps10i235* alignment in *L*. *japonicus* and *M*. *truncatula*, the R3 mediated homologous recombination could possibly give rise to the deletion of the repeat flanking region in *M*. *truncatula* and *L*. *japonicus*. Therefore, it is very likely that the insertion of the mitovirus sequence happened in the mitogenome of the common ancestor of the eight species of the Faboideae, and which, is followed by lineage specific loss of the insertions mediated by short repeats. The other two introns (*rps3i74*, and *nad2i709*) also showed length variations (S2 Fig), whereas no direct repeats or deletion related repeats were found in them, and indicated either quick sequence erosions followed by sequence deletions or inadequate lineage sampling or both.

### Nucleotide substitution rates

Plant mitogenomes are well known for slow evolving genes with synonymous nucleotide substitution rate 3–5 times lower than that of the chloroplast, and 10–20 times lower than the nuclear genes [45]. Some plant mt genomes unexpectedly show significantly higher substitution rates, such as the mitogenomes of *Silene conica* and *S*. *noctiflora* [3]. The mutation pressure hypothesis advances negative correlation of genome size and mutation rates, whereas substitution rate estimations of the mt genomes in *Silene* species [3], species of the Curcubitaceae [4] and *Geranium* [46] suggested the opposite being true. The accumulation of eight mt genomes of nearly two-fold range of genome size variation in the subfamily Faboideae allowed us to test this hypothesis. Our analysis based on a concatenated 29 protein coding genes data set recovered similar patterns of divergence in both the synonymous and nonsynonymous trees (Fig 2; S3 Table). A ten-fold and a twenty-fold rates of variations were observed for nonsynonymous (d_N_) and synonymous (d_S_) substitution rates, respectively, which lead to a six-fold and a four-fold absolute substitution rate per billion years for nonsynonymous (R_N_) and synonymous (R_S_) substitution rates, respectively. The R_S_ of *S*. *japonica* is the lowest among eight Faboideae mt genomes, which is comparable to that of the *M*. *pinnata*, *G*. *max*, and *L*. *japonicus*, whereas only half of that of *V*. *radiata*, and one-fourth of that of *M*. *truncatula* and *V*. *angularis*. The smallest mt genome found in *M*. *truncatula* showed significantly higher substitution rates compared with the majority of the Faboideae mt genomes, whereas some large genomes can also evolve quickly, such as *V*. *angularis*. The high synonymous substitution rate observed in *V*. *angularis* could possibly be attributed to reduced gene conversion rate as suggested by Sloan et al. [3] however, without strong evidence presented. Because the predicted RNA editing site number (461) and the average intron length (1,788 bp) in *V*. *angularis* did not show distinctive reduction and were very close to that of *V*. *radiata* (468 sites and 1,788 bp), the latter has only one-half of the absolute synonymous substitution rate as that of *V*. *angularis*. Therefore, substitution rate analysis in the eight mt genomes suggested two important evolutionary forces underlying the mitogenomic evolution of the Faboideae. First, our study lends support to the mutation burden hypothesis as *S*. *japonica* with the largest mt genome in the Faboideae has the lowest R_S_, whereas *M*. *truncatula* with the smallest mt genome holds the highest R_S_. Second, the exceptionally high substitution rate observed in the average sized mitogenome of *V*. *angularis* argues for reduced gene conversion rate in this species.

### MtDNA sharing

**Fig 6:**
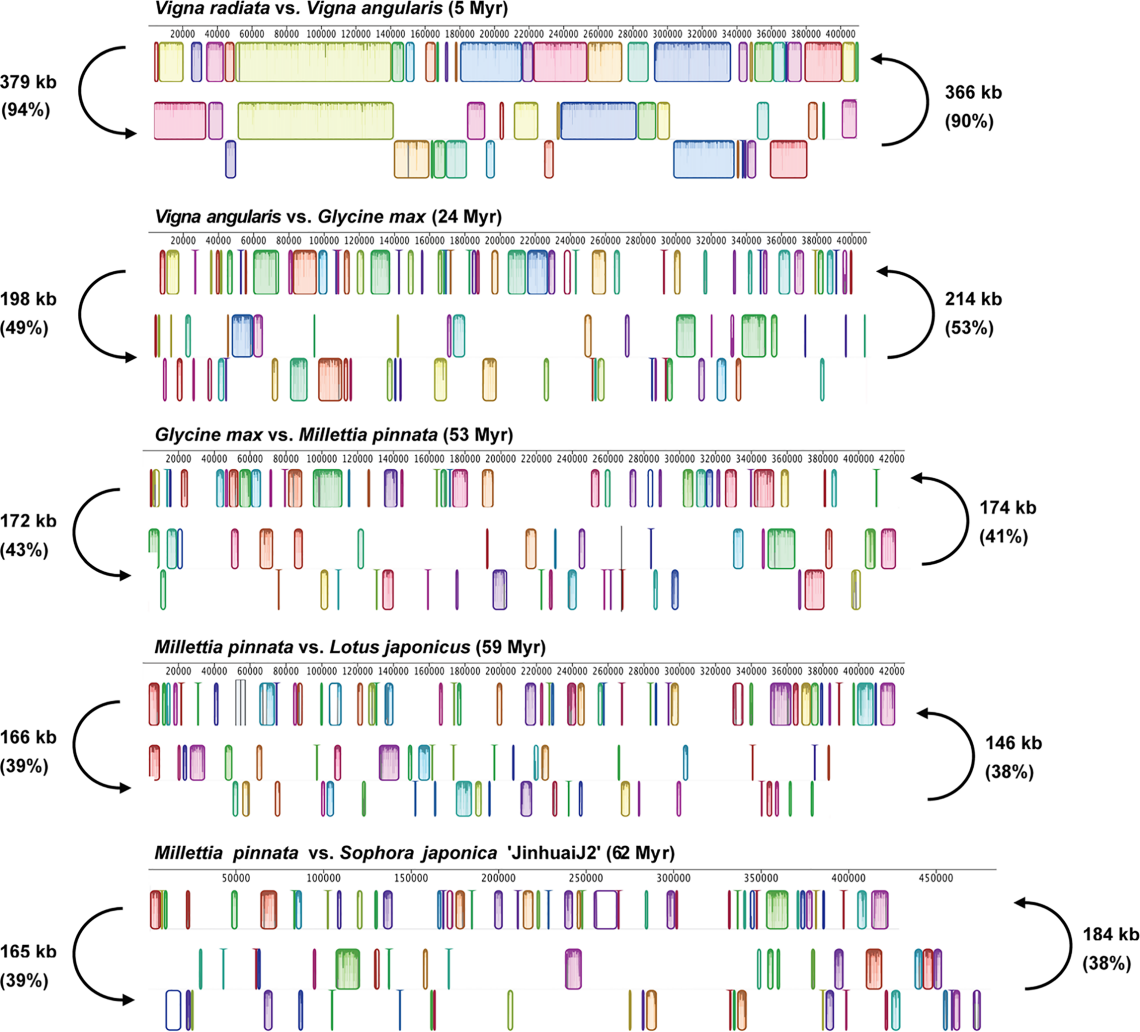
Shared mtDNA among Faboideae mitogenomes as shown by Mauve alignments of five pairs of Faboideae species at varying evolutionary depths.

We investigated mtDNA sharing in eight Faboideae mt genomes. The shared mtDNA amount is partially correlated with the divergence time of the species pair through the evolution of the Faboideae (Fig 6). The mt genomes of two different *Vigna* species share 366–379 Kb of their mtDNA sequence, constituting 90% of the genome sequence of *V*. *angularis* and 94% of that of the *V*. *radiata* with a divergence time of ca. 5 Myr. This shared amount declined drastically to 198–214 Kb, and accounted for 53% of the genome sequence of *G*. *max* and 49% of that of the *V*. *angularis* when the divergence time staggered to 24 Myr. The shared mtDNA amount further declined to 172–174 Kb, taking up 41% and 43% of the complete sequence of *M*. *pinnata* and *G*. *max*, respectively, when the divergence time was increased to 53 Myr. The shared mtDNA amount and related ratio continued to drop with the increase of the divergence time, whereas the shared amount appeared to undergo a moderate increase for *S*. *japonica* with 184 Kb shared sequence. This might be attributed to the 80 Kb (20%) genome size increase of *S*. *japonica* compared with that of the other Faboideae mitogenomes including *M*. *pinnata*. The amount of mtDNA sharing was also negatively correlated with the accumulation of species-specific sequences, such as *S*. *japonica* mitogenome contained 122 Kb anonymous sequences, and added up to 25% of its genome size. *M*. *pinnata*, *M*. *truncatula* and *L*. *japonicus* contained ca. 14% unique sequences, whereas the more derived *G*. *max* contained 9% and *Vigna* 5% of unique sequences, respectively.

### Repeats and genome structure

**Fig 7:**
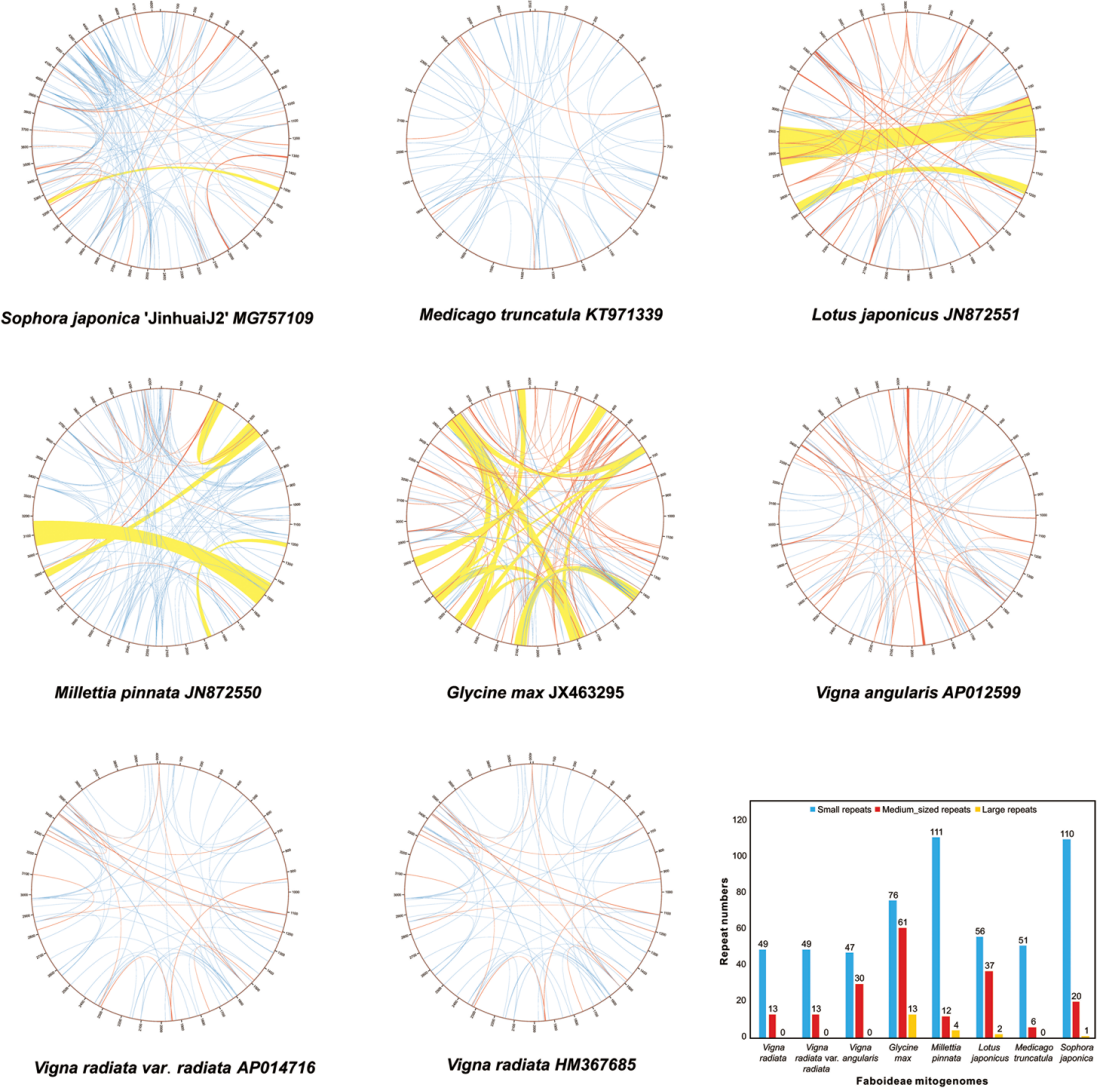
Repeat distributions and occurrences in the eight Faboideae mitogenomes. Large repeats ≥1,000 bp in length are indicated in orange, medium-sized repeats in the range of 100–1,000 bp in length are indicated in red, and small repeats <100 bp in length are colored blue.

As an important component of plant mt genomes, repeated sequences contribute much of the genome structure complexities through mediation of homologous recombinations [1]. To investigate the patterns of repeat distributions in the Faboideae, we sorted out all the repeat pairs with unique start and end coordinates from blastn output in three grades: large repeats (> = 1000 bp), medium-sized repeats (100–1000 bp) and small repeats (<100 bp). Repeat abundance and composition varied greatly in the Faboideae (Fig 7). *G*. *max* mitogenome has the highest repeat amount in the Faboideae in terms of repeat sequence coverage (72,873 bp, 18%) with 150 repeats including 61 medium-sized, and 13 large repeats. This is in stark contrast to *M*. *truncatula* with the lowest repeat amount in repeat sequence coverage (5,406 bp, 2%) with a total of 51 repeats including 6 medium-sized repeats but no large repeats. All the Faboideae mt genomes harbor relatively high numbers of small repeats (e.g., ranging from 47 repeat pairs in *V*. *angularis* to 110 and 111 repeats in *S*. *japonica* and *M*. *pinnata*, respectively), whereas large repeats are limited in the Faboideae. Three *Vigna* species mt genomes and the mt genome of *M*. *truncatula* have no large repeats and the *S*. *japonica* mt genome contained only one large repeat pair of 2,160 bp (Table 2); *L*. *japonicus* contained two; and *M*. *pinnata* had four. As large repeats tend to recombine more frequently than medium-sized repeats with moderate recombination rates and small repeats with minor recombination activity, there is a positive correlation of repeat length and recombination frequency [2]. The mt genome of *G*. *max* is notoriously difficult to assembly due to the presence of a large number of recombinationally active repeats, which give rise to a complex molecular pool of 760 circles. However, no recombination activity was detected in the mt genome of *V*. *radiata* [20]. *S*. *japonica* mt genome has a repeat compositional pattern somewhat intermediate to the mt genome of recombinationally active *G*. *max* and the recombinationally quiescent *V*. *radiata*. However, it is similar to the repeat content in *V*. *angularis*, which, mediates moderate recombinations with rates for ten medium-sized repeats ranging from 0.07 to 0.24, and two small repeats being 0.05 and 0.11, respectively [19]. *S*. *japonica* mitogenome might likely have a recombination system similar to *V*. *angularis* and that at least some repeats are recombinationally active. Homologous recombination products were detected for the longest repeats of 2,160 bp in two out of six reads spanning the total length of the repeats given that our PacBio long reads data had an average read coverage depth of just 15×. We further identified 25 pseudogene fragments ranging from 28 to 182 bp in the intergenic spacers of *S*. *japonica* that matched ten protein coding genes (*atp1*, *atp6*, *ccmC*, *cox2*, *nad1*.*x5*, *nad2*.*x5*, *nad6*, *nad9*, *rpl5*, *rps4*) with identities ranging from 92% to 100%. Blastn search of all these pseudogene fragments against the NCBI nucleotide database yielded much lower similarities with any other species than *S*. *japonica*, thus ruling out the possibilities of the origin of these pseudogenes from horizontal gene transfers from other plants. The two largest pseudogene fragments of 331 bp and 201 bp (pseudo-*rpl5* and pseudo-*atp6*) were located in two different sets of the repeats with their corresponding functional protein genes, and indicated the origins of these two pseudogene fragments from repeat recombinations.

**Table 2:** Distribution of repeats (>100 bp) in the mitochondrial genome of *Sophora japonica* ‘JinhuaiJ2’.

No.	Identity	Copy-1		Copy-2		Size (bp)	Type^a^
		Start	End	Start	End		
Ra	100.00	150,477	152,636	323,868	326,027	2,160	DR
Rb	99.87	131,028	131,822	199,473	198,679	795	IR
Rc	98.21	47,599	48,040	387,922	387,477	446	IR
Rd	99.05	36,092	36,406	470,057	470,371	315	DR
Re	89.42	313,081	313,526	346,495	346,051	463	IR
Rf	99.14	142,655	142,886	323,014	322,783	232	IR
Rg	99.54	262,719	262,934	336,368	336,152	217	IR
Rh	86.96	123,745	124,024	433,969	433,672	299	IR
Ri	100.00	326,022	326,181	409,770	409,929	160	DR
Rj	100.00	110,865	111,010	215,167	215,312	146	DR
Rk	98.52	9,508	9,642	287,854	287,988	135	DR
Rl	100.00	342,433	342,559	395,427	395,301	127	IR
Rm	96.83	144,415	144,540	176,333	176,208	126	IR
Rn	99.15	46,875	46,991	280,763	280,647	117	IR
Ro	98.25	249,100	249,213	342,464	342,575	114	DR
Rp	96.43	346,889	346,999	359,834	359,723	112	IR
Rq	98.02	413,382	413,481	427,948	428,048	101	DR
Rr	95.33	103,082	103,184	369,910	369,804	107	IR
Rs	92.16	15,032	15,133	214,266	214,165	102	IR
Rt	86.49	27,117	27,226	387,922	387,813	111	IR
Ru	79.86	143,849	143,965	279,630	279,773	144	DR

Notes

^a^DR or IR: direct or inverted repeats, respectively.

**Fig 8:**
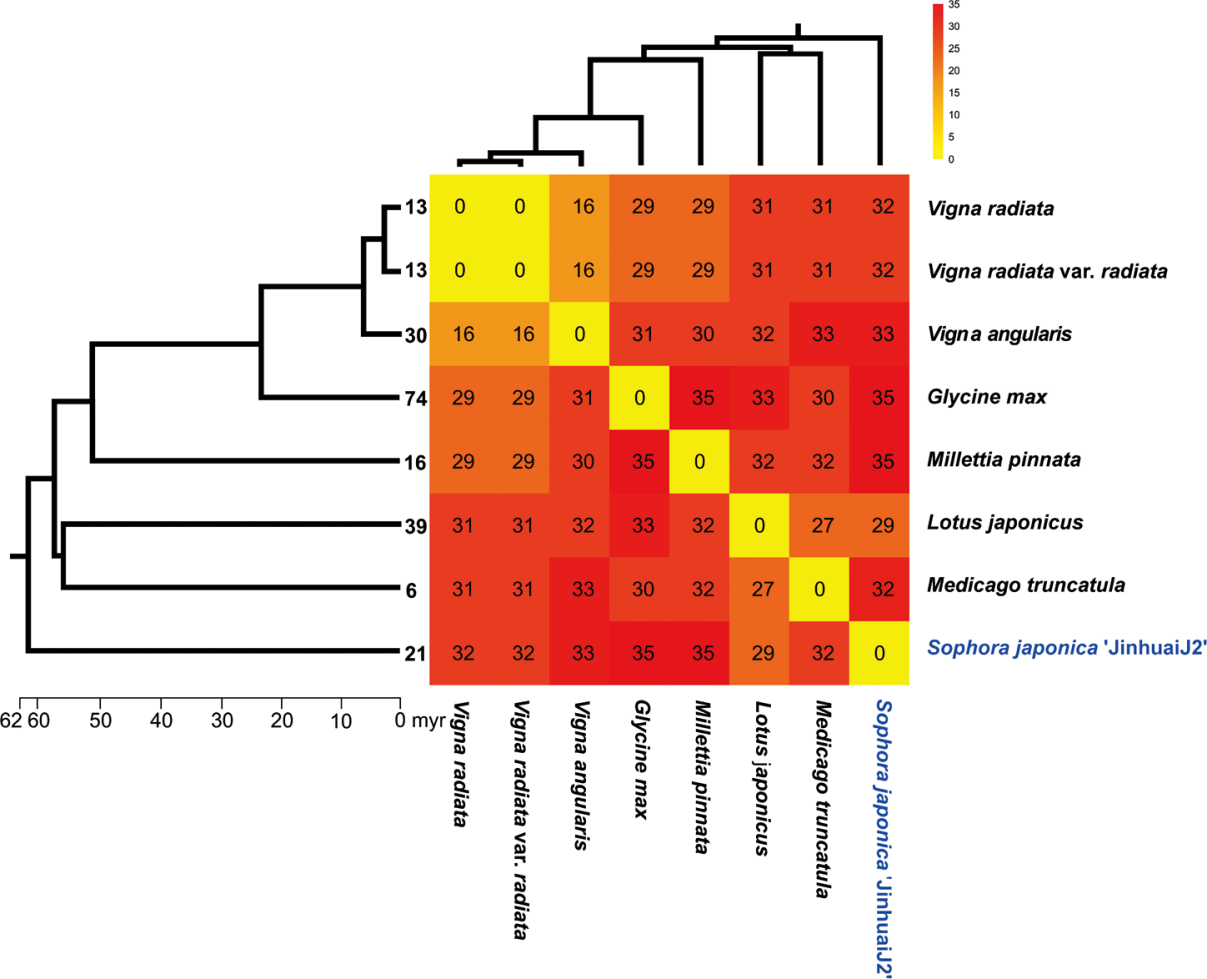
Heatmap of mitogenome rearrangements in pairwise comparison of species in the Faboideae along a phylogenetic tree based on topologies constrained according to version 13 of the Angiosperm phylogeny website (http://www.mobot.org/mobot/research/apweb/.) with divergence time estimated from Timetree website (http://www.timetree.org).

Analysis of gene arrangements of mt genomes in the Faboideae revealed distinctive patterns of genome structure dynamics (Fig 8). The number of genome rearrangement times is positively correlated with the divergence time of the specific species pair. We further mapped the number of medium-sized and large repeats on the branch tips and found correlation of rearrangement times with repeat abundance. For the mt genome of *G*. *max* (branch age: 24 Myr) with most abundant repeats, an average of 32 rearrangements were needed to reconcile its mt genome with any other mt genome of the Faboideae. This is the same with that of the mt genomes of *M*. *pinnata* and *S*. *japonica* with a divergence time of 53 Myr and 62 Myr. However, for the mt genomes of the deep branched *L*. *japonicus* and *M*. *truncatula* with limited repeat contents, an average of 30 rearrangements were needed to align each of their genomes with any other mt genome of the Faboideae. Our study of the conserved gene clusters in the Faboideae provided additional evidence for the dynamic nature of *G*. *max* mitogenome (S4 Table). All the Faboideae mt genomes have the *rps19*–*rps3* gene cluster that could be dated to the endosymbiont ancestor with the exception of that of the *G*. *max*. The mitogenome of *G*. *max* also exclusively lost the gene cluster of *atp4*–*nad4L* that is widely distributed in angiosperm mt genomes. However, *S*. *japonica* mitogenome has a relatively complete set of conserved mt gene clusters with the exception of the seed plant originated gene cluster of *rpl5*–*rps14*–*cob* and eudicots specific gene cluster of *trnEttc*–*trnMcat-cp*, which indicated the lineage specific loss of the gene cluster after its divergence from the rest of the Faboideae members.

## Conclusions

We produced a high quality assembly of the mt genome of *S*. *japonica* and compared it with the other seven available mt genomes of the Faboideae. The gene content of *S*. *japonica* mt genome is similar to that of the other Faboideae mt genomes with the exception of a few gene losses and gains. Despite similar intron content across Faboideae mt genomes, four out of 17 cis-spliced introns showed distinctive length variations among these mitogenomes, which likely could be due to the recombinational activity of the small repeats measuring a few bases and reflected the significance of small repeats in the mitogenomic sequence evolution of the Faboideae. *S*. *japonica* mt genome showed the lowest absolute synonymous substitution rates in contrast to the highest for *M*. *truncatula* and *V*. *angularis*, and implied that two important evolutionary forces underlined the evolutionary pattern of Faboideae mitogenomes. MtDNA sharing amount analyses revealed the negative correlation between divergence time of the species pair and the shared amount in the Faboideae, and mirrored the positive correlation between divergence time and the accumulation of species-specific sequence amount. Repeat distribution patterns varied greatly among Faboideae mt genomes. The repeat compositional pattern, along with the observation of the recombination evidence for the longest repeats, as well as the detection of pseudogene fragments in the intergenic spacers in relatively repeat depauperate mt genome of *S*. *japonica*, suggested the importance of repeat recombinations in the structural evolution of the Faboideae as exemplified by the dynamic mitogenomic structure rearrangements.

## Supporting information

S1 Fig Phylogeny of eight Faboideae mitochondrial genomes with *Cucurbita pepo* as an outgroup. Numbers above each node represent bootstrap values from 1000 replicates. Branch lengths are in units of synonymous substitutions per synonymous site.
(TIF)

S2 Fig Schematic demonstration of the two protein gene alignments (*nad2* and *rps3*) showing intron length variations and the positions of the intron-positioned direct repeats. (TIF)

S1 Table Sequencing details of the current study. (DOCX)

S2 Table Comparison of the cis-spliced intron length (bp) in the Faboideae mitogenomesa. (DOCX)

S3 Table Synonymous and nonsynonymous substitution rates. (DOCX)

S4 Table Conserved mitochondrial gene clusters and its distribution in Faboideae. (DOCX)
