# Supplementary figures and images for: Assembly and comparative analysis of the complete mitochondrial genome sequence of Sophora japonica ‘JinhuaiJ2’
Source: PLoS One. 2018 Aug 16;13(8):e0202485. doi: 10.1371/journal.pone.0202485 (PMC6095553; doi:10.1371/journal.pone.0202485)

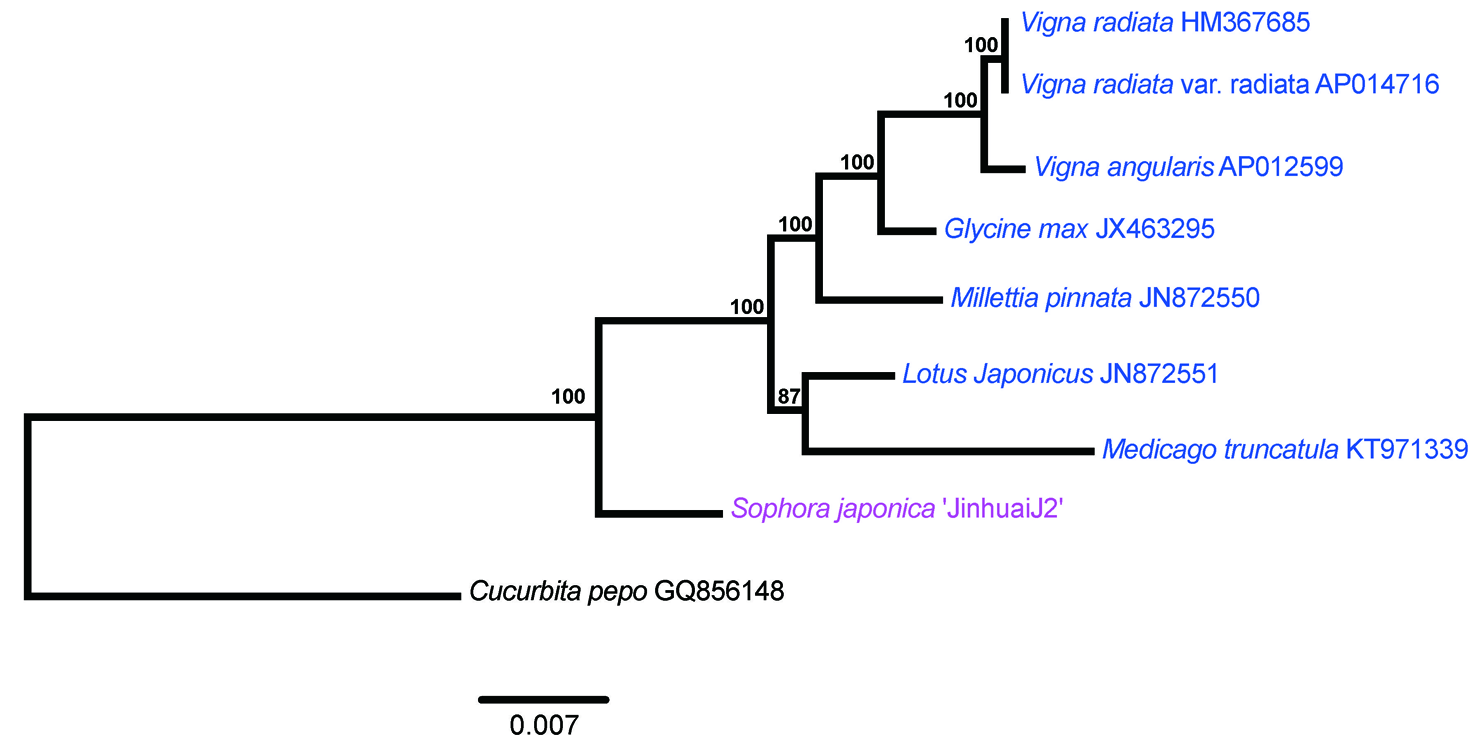

Supplement: S1 Fig — Numbers above each node represent bootstrap values from 1000 replicates. Branch lengths are in units of synonymous substitutions per synonymous site. (TIF) [file pone.0202485.s001.tif]

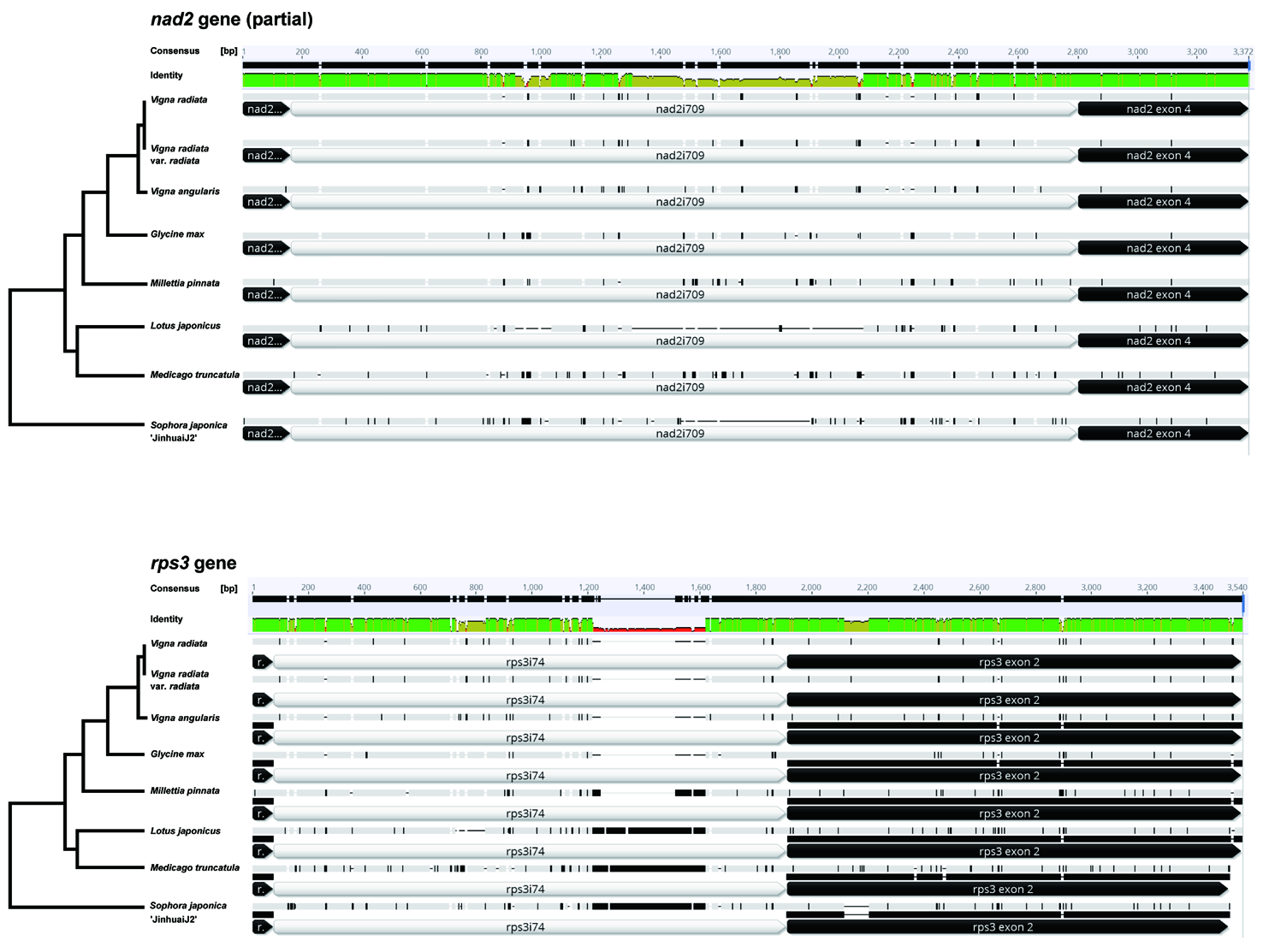

Supplement: S2 Fig — (TIF) [file pone.0202485.s002.tif]
