# Supplementary material for: Assembly and comparative analysis of the complete mitochondrial genome sequence of Sophora japonica ‘JinhuaiJ2’
Source: PLoS One. 2018 Aug 16;13(8):e0202485. doi: 10.1371/journal.pone.0202485 (PMC6095553; doi:10.1371/journal.pone.0202485)
Supplement: S1 Table — (DOCX) [file pone.0202485.s003.docx]

|  | Total reads count | Total reads bases | Mt reads count | Mt reads bases (ratio) |
| --- | --- | --- | --- | --- |
| Sequencing Technology |  |  |  |  |
| Illumina Hiseq 2000 | 33,775,098 | 5,066,264,700 | 1,176,183 (3.48%) | 176,427,450 (3.48%) |
| PacBio RSII Sequencing | 3,049 | 38,229,846 | 1,104 (36.21%) | 15,884,959 (41.55%) |

Note: (Mt) Mitochondrial
