# Supplementary material for: Assembly and comparative analysis of the complete mitochondrial genome sequence of Sophora japonica ‘JinhuaiJ2’
Source: PLoS One. 2018 Aug 16;13(8):e0202485. doi: 10.1371/journal.pone.0202485 (PMC6095553; doi:10.1371/journal.pone.0202485)
Supplement: S2 Table — (DOCX) [file pone.0202485.s004.docx]

|  | ccmFci829 | nad1i477 | nad1i728 | nad2i156 | nad2i709 | nad2i1282 | nad4i461 | nad4i976 | nad4i1399 | nad5i230 | nad5i1872 | nad7i140 | nad7i209 | nad7i676 | nad7i917 | rps3i74 | rps10i235 |
| --- | --- | --- | --- | --- | --- | --- | --- | --- | --- | --- | --- | --- | --- | --- | --- | --- | --- |
| VraI | 4,090 | 1,469 | 3,167 | 1,262 | 2,606 | 1,516 | 1,444 | 3,335 | 2,586 | 877 | 942 | 914 | 1,352 | 1,068 | 2,043 | 1,397 | 2,875 |
| VraII | 4,090 | 1,469 | 3,167 | 1,262 | 2,606 | 1,516 | 1,444 | 3,335 | 2,586 | 877 | 942 | 914 | 1,352 | 1,068 | 2,043 | 1,397 | 2,875 |
| Van | 4,090 | 1,469 | 3,167 | 1,261 | 2,610 | 1,515 | 1,444 | 3,244 | 2,583 | 872 | 936 | 914 | 1,352 | 1,068 | 2,059 | 1,403 | 2,890 |
| Gma | 4,101 | 1,451 | 3,164 | 1,250 | 2,593 | 1,516 | 1,438 | 3,345 | 2,603 | 863 | 942 | 916 | 1,339 | 1,064 | 2,053 | 1,387 | 2,883 |
| Mpi | 4,074 | 1,455 | 3,159 | 1,246 | 2,616 | 1,517 | 1,433 | 3,357 | 2,076 | 859 | 942 | 919 | 1,343 | 1,059 | 2,075 | 1,522 | 2,480 |
| Mtr | 958 | 1,446 | 3,175 | 1,340 | 2,162 | 1,514 | 1,432 | 3,339 | 2,608 | 903 | 942 | 914 | 1,341 | 1,055 | 2,032 | 1,802 | 924 |
| Lja | 964 | 1,450 | 3,158 | 1,244 | 1,714 | 1,520 | 1,441 | 3,345 | 2,603 | 876 | 947 | 919 | 1,342 | 1,059 | 2,084 | 1,778 | 973 |
| Sja | 952 | 1,439 | 3,142 | 1,233 | 2,552 | 1,505 | 1,423 | 3,306 | 2,801 | 844 | 930 | 910 | 1,333 | 1,054 | 2,011 | 1,748 | 2,773 |

Notes:

^a^ (VraI) *Vigna radiata*; (VraII) *Vigna radiata* var. *radiata*; (Van) *Vigna angularis*; (Gma) *Glycine max*; (Mpi) *Millettia pinnata*; (Mtr) *Medicago truncatula*; (Lja) *Lotus japonicus*; (Sja) *Sophora japonica* ‘JinhuaiJ2’.
