# Supplementary material for: Assembly and comparative analysis of the complete mitochondrial genome sequence of Sophora japonica ‘JinhuaiJ2’
Source: PLoS One. 2018 Aug 16;13(8):e0202485. doi: 10.1371/journal.pone.0202485 (PMC6095553; doi:10.1371/journal.pone.0202485)
Supplement: S3 Table — (DOCX) [file pone.0202485.s005.docx]

|  | Time | d_N_ | R_N_ | d_S_ | R_S_ |  |
| --- | --- | --- | --- | --- | --- | --- |
| Branch | (Ma) | (sub/site) | (sub/site/Ga) | (sub/site) | (sub/site/Ga) | d_N_/d_S_ |
| *Vigna radiata* | 5 | 0.00087 | 0.1740 | 0.00126 | 0.2520 | 0.6904 |
| *Vigna radiata* var. *radiata* | 5 | 0.00087 | 0.1740 | 0.00126 | 0.2520 | 0.6904 |
| *Vigna angularis* | 5 | 0.00128 | 0.2560 | 0.00231 | 0.4620 | 0.5541 |
| *Glycine max* | 24 | 0.00107 | 0.0445 | 0.00345 | 0.1437 | 0.3101 |
| *Millettia pinnata* | 53 | 0.00403 | 0.0760 | 0.00740 | 0.1396 | 0.5445 |
| *Lotus japonicus* | 59 | 0.00439 | 0.0744 | 0.00892 | 0.1511 | 0.4921 |
| *Medicago truncatula* | 59 | 0.00989 | 0.1676 | 0.02369 | 0.4015 | 0.4174 |
| *Sophora japonica* ‘JinhuaiJ2’ | 62 | 0.00437 | 0.0705 | 0.00794 | 0.1282 | 0.5503 |
