# Supplementary material for: Assembly and comparative analysis of the complete mitochondrial genome sequence of Sophora japonica ‘JinhuaiJ2’
Source: PLoS One. 2018 Aug 16;13(8):e0202485. doi: 10.1371/journal.pone.0202485 (PMC6095553; doi:10.1371/journal.pone.0202485)
Supplement: S4 Table — (DOCX) [file pone.0202485.s006.docx]

| No. | Gene clusters | Origin | Distribution in Faboideae^a^ |
| --- | --- | --- | --- |
| 1 | rps3−rpl16 | Bacteria | All |
| 2 | rps19−rps3 | Bacteria | All except *Glycine* |
| 3 | rrn18−rrn5 | Bacteria | All |
| 4 | trnfM*cat*−rrn26 | Streptophytes | All |
| 5 | cox3−sdh4 | Seed plants | All |
| 6 | atp8−cox3 | Seed plants | *Millettia*, *Lotus*, and *Sophora* |
| 7 | nad3−rps12 | Seed plants | All |
| 8 | rpl5−rps14−cob | Seed plants | All except *Sophora* |
| 9 | rps10−cox1 | Seed plants | All except *Millettia* |
| 10 | atp4−nad4L | Angiosperms | All except *Glycine* |
| 11 | trnSgct−trnFgaa | Angiosperms | *Sophora*, *Lotus*, *Millettia*, and *Glycine* |
| 12 | trnFgaa−trnPtgg | Angiosperms | All |
| 13 | trnCgca−trnNgtt-cp−trnYgta | Eudicots | *Sophora*, *Lotus*, *Medicago*, and *Millettia* |
| 14 | trnEttc−trnMcat-cp | Eudicots | All except *Sophora* |
| 15 | trnGgcc−trnQttg | Eudicots | All |
| 16 | <trnDgtc-cp><nad4> | Eudicots | All |

Notes:

^a^ The distributions are based on eight available mitochondrial genomes: *Sophora japonica* ‘JinhuaiJ2’ MG757109, *Medicago truncatula* KT971339, *Lotus japonicus* JN872551, *Millettia pinnata* JN872550, *Glycine max* JX463295, *Vigna angularis* AP012599, *Vigna radiata* HM367685, and *Vigna radiata* var. *radiata* NM92 AP014716
